# Global distribution and evolution of nine major non-polio enteroviruses revealed by genomic data mining

**DOI:** 10.1016/j.bbrep.2026.102485

**Published:** 2026-02-09

**Authors:** Han Mo, Hui Li, Jiadong Wu, Liu Yi, Fenglan He, Qingmei Huang, Xian Zhang, Qian Yang, Tianmu Chen, Xianfeng Zhou

**Affiliations:** a Cancer Research Center, Jiangxi University of Chinese Medicine, Nanchang, China; b Jiangxi Provincial Health Commission Key Laboratory of Pathogenic Diagnosis and Genomics of Emerging Infectious Diseases, Nanchang Center for Disease Control and Prevention, Nanchang, China; c State Key Laboratory of Molecular Vaccinology and Molecular Diagnostics, School of Public Health, Xiamen University, Xiamen, China; d National Key Laboratory of Intelligent Tracking and Forecasting for Infectious Diseases (NITFID), National Institute for Viral Disease Control and Prevention, Chinese Center for Disease Control and Prevention, Beijing, 102206, China; e National Polio Laboratory, World Health Organization Polio Reference Laboratory for the Western Pacific Region, National Institute for Viral Disease Control and Prevention, Chinese Center for Disease Control and Prevention, 155 Changbai Road, Beijing, 102206, China; f National Health Commission Key Laboratory of Laboratory Biosafety, National Institute for Viral Disease Control and Prevention, Chinese Center for Disease Control and Prevention, 155 Changbai Road, Beijing, 102206, China

**Keywords:** Enteroviruses, Epidemic trends, Evolutionary dynamics, Selection pressure, Capsid proteins, Molecular surveillance

## Abstract

**Objectives:**

This study aimed to elucidate the global epidemic trends and evolutionary characteristics of nine major non-polio enterovirus serotypes (CVA2, CVA4, CVA6, CVA10, CVA16, CVB3, CVB5, EV-A71, and EV-D68) through genomic data mining, focusing on their spatiotemporal distribution and evolutionary dynamics.

**Design:**

We employed a data mining framework integrating programming, phylogenetic analysis, Bayesian evolutionary modeling, and selection pressure assessment. Over 40,000 genomic sequences from GenBank were analyzed to reconstruct temporal phylogenies, estimate evolutionary rates, and characterize amino acid variability in the capsid protein VP1. Seasonal decomposition and spatial-temporal trend modeling were applied to evaluate epidemic patterns across the six WHO regions.

**Results:**

Key findings include [1]: Distinct biennial or triennial epidemic cycles for EV-D68 and clear seasonal peaks for HFMD-associated serotypes [2]; A preliminary observation termed the “60% Transcendence” phenomenon, where once cumulative VP1 nucleotide mutations reach approximately 60%, the cumulative non-synonymous amino acid mutations begin to exceed this threshold [3]; Evidence of episodic positive selection at critical VP1 codons, suggesting immune-driven evolution [4]; Divergent trends in relative genetic diversity, with EV-A71, CVA16, and CVA6 showing sustained expansion, while the diversity of CVB5 and EV-D68 declined sharply during the COVID-19 pandemic.

**Conclusions:**

This study provides valuable insights into the changing landscape of global enterovirus infections and underscores the critical role of genomic epidemiology in tracking their spread. Sustained research in this field is essential for developing effective strategies to prevent and control enterovirus-related diseases worldwide.

## Highlights

• Study shows spatiotemporal shifts of 9 enteroviruses across 6 WHO regions: EV-A71/CVA16/CVB5 in Asia/Europe, EV-D68 in Americas/Europe.
• Analyzed >40k genomes of 9 non-polio enteroviruses for phylogenies, evolutionary rates & capsid protein variability.
• New framework: 60% VP1 mutation rate triggers NSAM surge in antigenic loops (BC/DE/EF/GH/HI), revealing immune escape pressure.
• Episodic diversifying selection in VP1 loops necessitates genomic surveillance to track evolution & guide enterovirus vaccine design.

## Graphical abstract

**Figure:**
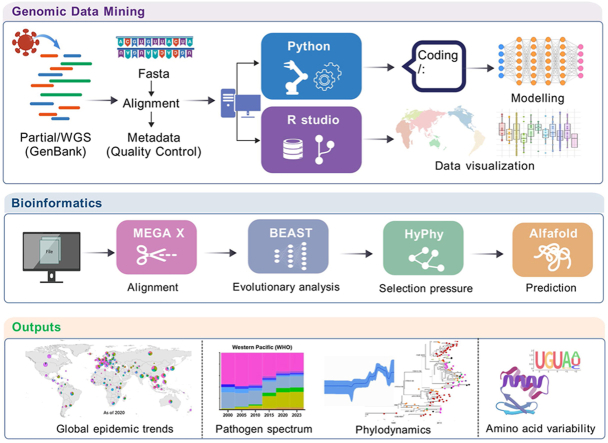

## 1 Background

Enteroviruses are a group of RNA viruses belonging to the *Picornaviridae* family, responsible for a wide range of human illnesses including hand, foot, and mouth disease (HFMD), viral myocarditis, and respiratory and neurological infections [1]. The enterovirus genome consists of a single-stranded positive-sense RNA molecule approximately 7.5 kilobases in length [2]. It is organized into two open reading frames (ORFs): the long ORF encodes a polyprotein, while the upstream ORF (uORF) is involved in viral replication within intestinal epithelial cells [3]. The polyprotein is subsequently cleaved by viral proteases to produce individual structural proteins (VP1-VP4) and non-structural proteins (2A–2C, 3A–3D), which are crucial for viral replication, assembly, and pathogenesis [2]. Human enteroviruses are classified into four species: enterovirus A, B, C, and D. Enterovirus D68 (EV-D68), a member of species D, has emerged as a significant pathogen of global concern in recent years [4,5]. The primary serotypes associated with HFMD include Coxsackievirus A (CVA) 4–7, 9, 10, and 16, Coxsackievirus B (CVB) 1–3 and 5, and Enterovirus A71 (EV-A71) [6]. Among these, CVA2, CVA4, CVA6, CVA10, CVA16, EV-A71, CVB3, CVB5, and EV-D68 have been identified as major contributors to global epidemics of HFMD, viral myocarditis, and respiratory illness, imposing a substantial disease burden.

Notably, EV-A71, CVA16, CVA6 and CVA10 have alternately dominated the landscape of HFMD-associated enterovirus infections, resulting in thousands of fatalities over the past 20 years [7,8]. Continuous monitoring of the distribution and evolution of these serotypes is crucial for understanding the dynamic nature of these infections and for developing effective prevention and control strategies. Such understanding, particularly of their evolutionary profiles, is essential for predicting outbreaks and implementing timely interventions to mitigate disease spread. Indeed, the dominance of specific serotypes in different regions underscores the importance of genomic epidemiology. Therefore, genomic epidemiology plays a vital role in tracking viral transmission, identifying potential outbreak hotspots, and elucidating the evolutionary features of major enterovirus serotypes [[7], [8], [9]].

In this study, we performed large-scale genomic data mining using sequences available in GenBank to analyze the global epidemic trends and evolutionary features of nine major serotypes of enteroviruses mentioned above. By analyzing the spatiotemporal distribution and etiological characteristics of enteroviruses, we identified critical inflection points in the prevalence of different serotypes, highlighting the need for persistent surveillance. This work provides important insights into the evolving epidemiology of enteroviruses and reaffirms the value of genomic epidemiology in tracking their global spread. Sustained research in this field is essential for formulating effective strategies to mitigate the burden of enterovirus-related diseases worldwide.

## 2 Material and methods

### 2.1 Sequence downloading for each serotype

All available gene sequences for each target serotype were downloaded from the NCBI Virus database (https://www.ncbi.nlm.nih.gov/labs/virus/vssi/) up to 2023. After filtering out potential duplicates (e.g., clones), a total of 836 (CVA2), 1601 (CVA4), 12,158 (CVA6), 3069 (CVA10), 7987 (CVA16), 15,176 (CVB3), 1550 (CVB5), 2585 (EV-A71), and 5752 (EV-D68) sequences, along with their metadata, were obtained. Complete or near-complete genome sequences and complete VP1 gene sequences were used for subsequent phylogenetic analyses. WHO region classifications (https://ourworldindata.org/grapher/who-regions) were used to analyze the global pathogen spectrum fluctuations.

### 2.2 Trend analysis and seasonal decomposition

Trend analysis was performed using time series decomposition. The seasonal decomposition procedure from the statsmodels.tsa.seasonal module (version 0.14.2) in Python (version 3.12.4) was applied. An additive model was used to decompose the time series data into trend, seasonal, and residual components.

### 2.3 Bayesian evolutionary analysis using BEAST

The global evolutionary dynamics of each serotype were inferred based on the complete VP1 region. A Markov chain Monte Carlo (MCMC) method implemented in BEAST (v1.8.4) was used to estimate divergence times, temporal phylogenies and evolutionary rates [10]. Analyses were conducted under a strict molecular clock model and a Bayesian skyline population growth model. The MCMC chain was run for 100 million generations to ensure convergence of all parameters. Output was analyzed using TRACER (v1.7.1) (http://beast.community/tracer), with effective sample size (ESS) values > 200 indicating convergence. A maximum clade credibility (MCC) tree was constructed using TreeAnnotator after discarding the first 10% of trees as burn-in, and the final tree was visualized using FigTree (v1.4.4).

### 2.4 Analysis of amino acid variability in VP1

Amino acid sequence logos were generated using WebLogo (https://weblogo.threeplusone.com). The VP1 protein structure was predicted using AlphaFold (https://alphafold.com/) and visualized with PyMOL (version 3.1). Amino acid sequences were aligned, and Shannon entropy values for each site were calculated using the online HIV LANL Entropy platform (https://hiv.lanl.gov/content/sequence/ENTROPY/entropy_one.html). Sites with an entropy value > 0.6 were considered highly variable.

### 2.5 Selection pressure analysis

Selection pressure was assessed by using the Mixed Effects Model of Evolution (MEME) implemented in the HyPhy software (version 2.0). Maximum Likelihood (ML) phylogenetic trees were first constructed using IQ-TREE (version 2.3.6). The optimal substitution model for each virus was selected via ModelFinder in IQ-TREE, and ML trees were built with 1000 bootstrap replicates. Episodic selection was analyzed using the internal branches method, with a p-value <0.1 considered indicative of episodic positive selection.

### 2.6 Graphics and statistical analysis

Geographical plots were generated using R (version 4.4.2) with packages including ggplot2, scatterpie, sf, etc. Heatmaps and other visualizations were produced using Python (version 3.12.4) with matplotlib and seaborn. Pearson's correlation coefficient was used to assess linear correlations between variables. All statistical tests were two-tailed, with p < 0.05 considered significant. Analyses were performed using GraphPad Prism 8.0.

## 3 Results

### 3.1 Global submission patterns of nine enterovirus serotypes

**Fig. 1:**
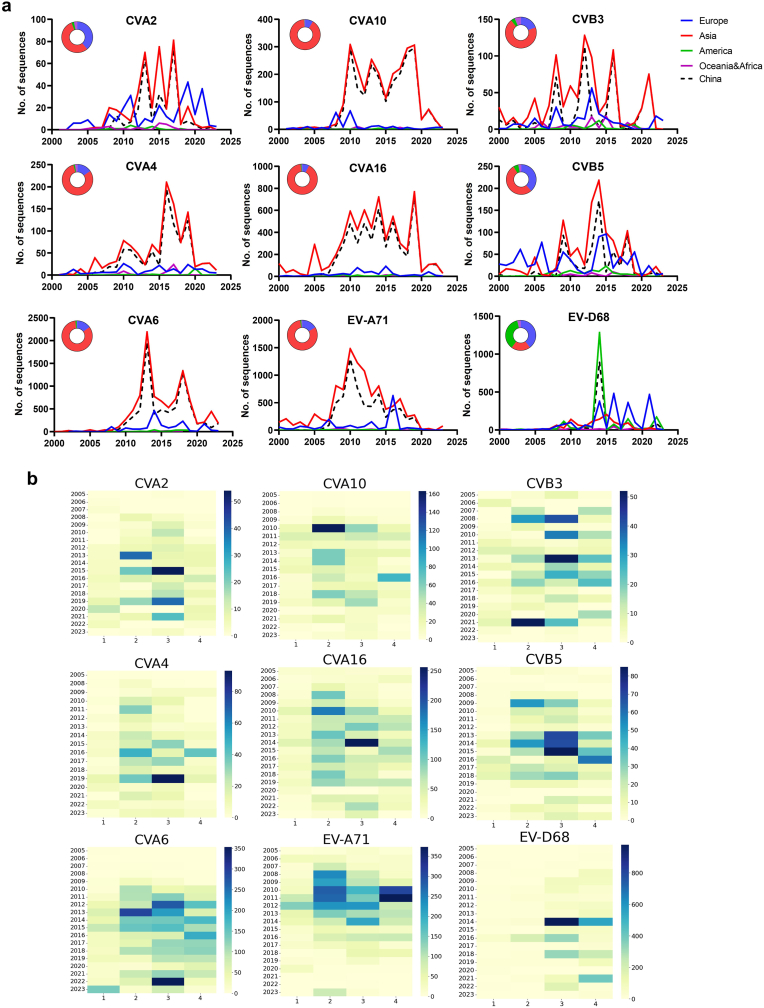
**Temporal trend of strain submissions of nine serotypes.** a) yearly strain submissions of each serotype from different continents; b) quarterly [[1], [2], [3], [4]] distribution of each serotype from 2005 to 2023. Data of Asia includes China, and data prior to 2005 was not included for quarterly distribution analysis due to the very limited submissions.

By analyzing strain submission data for nine serotypes from the GanBank, we examined metadata including collection date and country/region to investigate their continental-level distribution (Fig. 1a). The dynamic submission history for each serotype is shown in Videos 1–9. In general, most submissions originated from Asia and Europe, with China being a major contributor. Notably, EV-D68 outbreaks occurred in the Americas and Europe in 2014, followed by biennial epidemic waves. Overall, submission patterns reflected biennial or triennial cycles of enterovirus incidence (Fig. 1a). Quarterly distribution analysis revealed that most HFMD-associated strains were collected in the second and third quarters, consistent with the known seasonality of HFMD (Fig. 1b). In contrast, most EV-D68 strains were collected in the third and fourth quarters, aligning with the seasonality of respiratory infections (Fig. 1b). CVA6, which can cause atypical HFMD, was primarily collected in the second and third quarters during early outbreaks (2008–2013), but this pattern shifted to the third and fourth quarters after 2014. These submission patterns largely reflect regional diagnostic and research intensity, as well as underlying epidemic trends. Notably, submissions from China accounted for over 70% of the global total for these nine enteroviruses, indicating substantial public health focus on enterovirus-associated diseases in China over the past 15 years. This effort was spurred by the establishment of a national surveillance network following major EV-A71 outbreaks in 2008–2012, which caused thousands of deaths [11]. This network plays a crucial role in monitoring pathogen spectrum shifts and viral evolution nationwide [10,12]

Supplementary data related to this article can be found online at https://doi.org/10.1016/j.bbrep.2026.102485

### 3.2 Global pathogen spectrum fluctuation based on submission trends

**Fig. 2:**
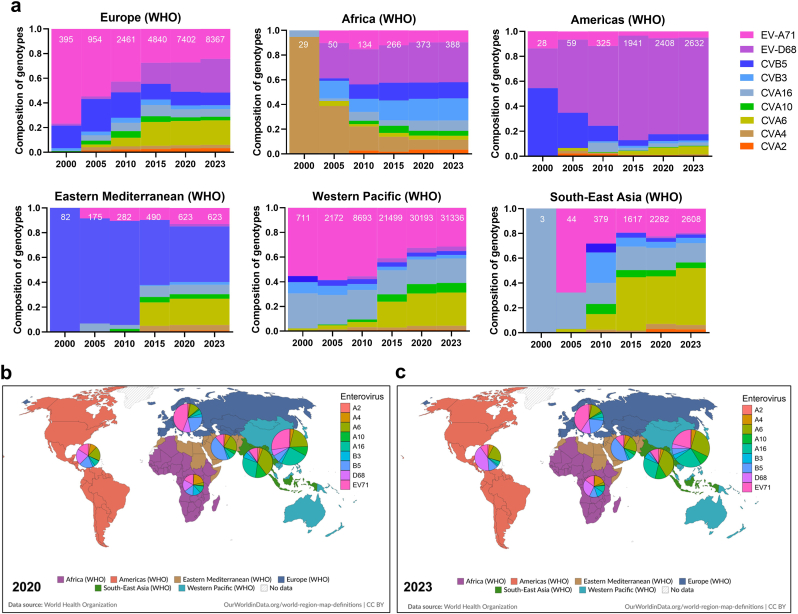
**A five-year interval time course of pathogen spectrum fluctuation of nine viruses in six WHO regions.** a) the first time point was set in 2000 as very limited submission by 2000. Dynamic composition of nine serotypes in six WHO regions. Composition of nine serotypes in six WHO regions by 2020 (b) and 2023 (c). Accumulated submission number was shown in the bar for each time point of the interval.

Globally, gene/genome submissions for the nine serotypes primarily came from Asia (20.4%–90.2%) and Europe (8.5%–34.7%) (Fig. 1a), mirroring the geographic hotspots of HFMD epidemics. We analyzed the temporal variation in the pathogen spectrum for CVA2, CVA4, CVA6, CVA10, CVA16, CVB3, CVB5, EV-A71, and EV-D68 across different countries and WHO regions. Since 2000, EV-A71 and CVA16 have dominated in the Western Pacific and South-East Asian regions (Supplementary Fig. S1). In Europe, CVB5 and EV-A71 were co-dominant during this period. By 2005, submissions from Eurasian countries had increased, with EV-A71, CVA16, and CVB5 remaining predominant. EV-D68 emerged as the dominant strain in North America, with sporadic reports elsewhere. Between 2005 and 2010, the proportion of EV-D68 increased significantly in North America, New Zealand, and several countries in Europe, Africa, and Asia (Supplementary Fig. S1). Notably, submissions for CVA6 increased rapidly from 2005 to 2010, peaking globally in 2011–2015, particularly in Europe, the Eastern Mediterranean, the Western Pacific, and Southeast Asia (Fig. 2a). Subsequently, CVA6 became one of the most dominant pathogens in these regions based on submission data. New Zealand exhibited a unique pathogen spectrum pattern compared to other Western Pacific countries (Fig. 2a). Between 2020 and 2023, minimal variation was observed in the pathogen spectrum at national or regional levels (Fig. 2b and c, Supplementary Fig. S1), suggesting either stable co-circulation of strains or reduced submission activity during the COVID-19 pandemic. Overall, the submission data revealed distinct spectral variations across the six WHO regions: Europe resembled the Western Pacific and South-East Asia, whereas Africa, the Americas, and the Eastern Mediterranean exhibited different patterns for CVA4, EV-D68, and CVB5 (Fig. 2a). Continuous submission of molecular surveillance data to public databases is essential to guide preventive measures and monitor global viral evolution.

### 3.3 Seasonality and epidemic trends

**Fig. 3:**
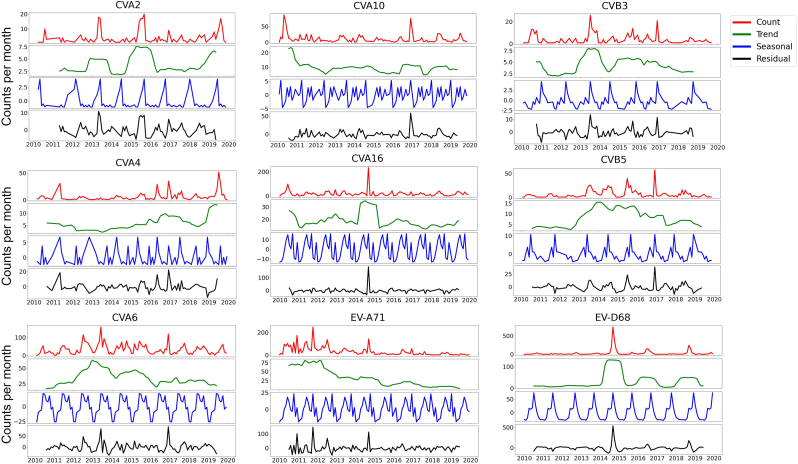
**Time series decomposition in trend, seasonality, and residuals of each serotype from 2010**–**2019.** Curves of count (red), trend (green), seasonal (blue) and residual (black) were displayed for each serotype.

EV-A71 and CVA16, two predominant HFMD-associated strains, exhibited similar trends and seasonality. Both showed high incidence before 2012, followed by a general decline, despite a submission peak in 2014 (Fig. 3). A similar trend was also observed for CVA10. Data fitting indicated comparable epidemic seasonality for these three viruses (Supplementary Fig. S2). Since 2012, CVA6 submissions increased sharply and remained highly variable until 2016, after which a steady decline continued until 2020 (Fig. 5, Supplementary Fig. S3). A rebound occurred thereafter. CVB3 and CVB5 showed trends similar to CVA6, although CVB5 did not rebound after 2020. EV-D68 exhibited a unique pattern, with outbreak waves in 2014, 2016, 2018, and 2022. Notably, the biennial peak was absent in 2020, coinciding with strict non-pharmaceutical interventions (NPIs) implemented during the COVID-19 pandemic (Supplementary Fig. S3). Similar attenuation of trends was observed for other viruses during this period, indicating that global NPIs significantly impacted enterovirus surveillance and testing.

**Fig. 4:**
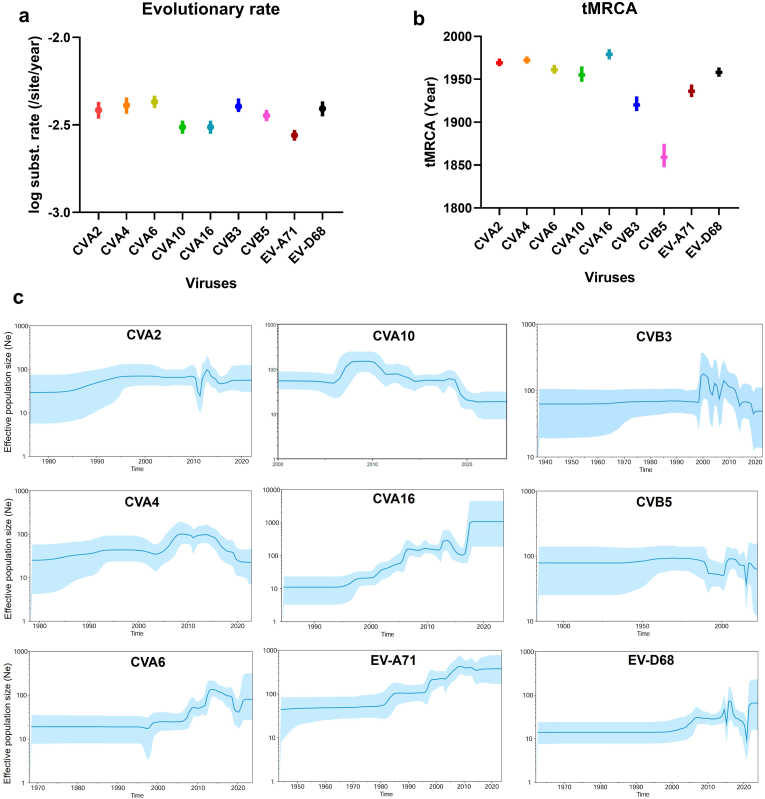
**Evolutionary characteristics of nine serotypes with Bayesian evolutionary analysis.** a) Estimation of evolutionary rate of nine serotypes; b) The tMRCAs of nine serotypes; c) Bayesian skyline plots of viral relative genetic diversity of each serotype. Bar means 95% HPD (highest posterior density). The light blue shadow means 95% CI. Statistical uncertainty in the tMRCA calculations was estimated as 95% HPD intervals.

**Fig. 5:**
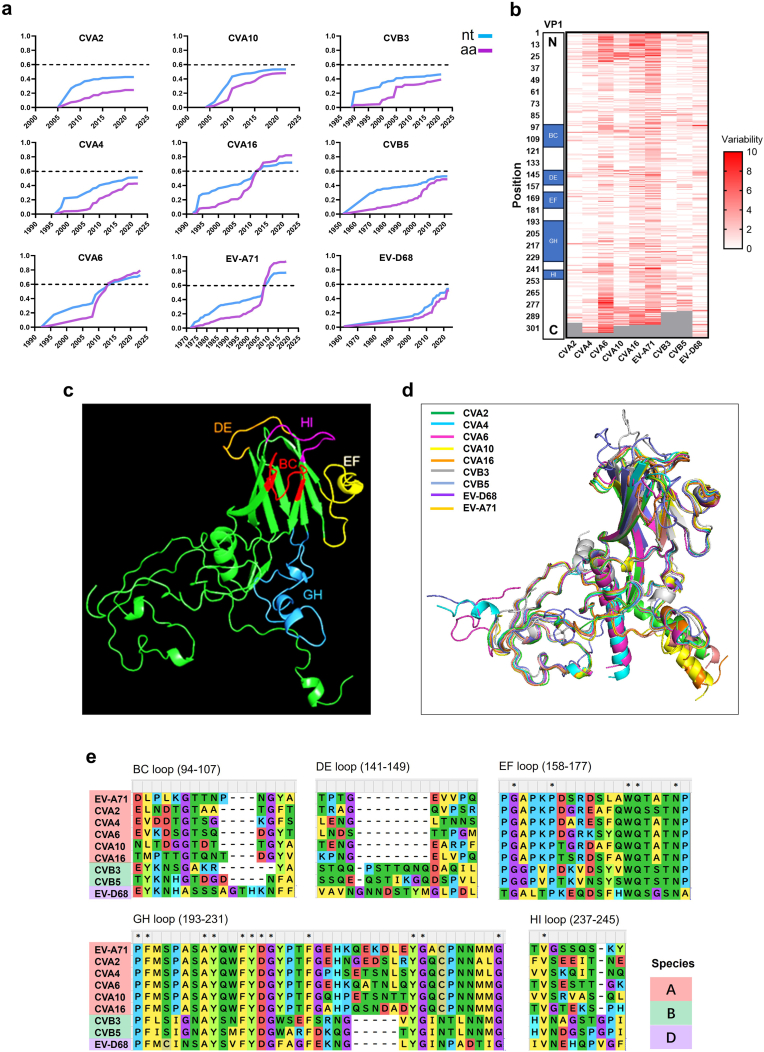
**Trends of cumulative nucleotide (nt) and non-synonymous amino acid (aa) mutation rate for each serotype** (a) and amino acid variabilities of VP1 of each serotype (b). Loops are labeled with different colors in the predicted structure of EV-A71 VP1 (c). The VP1 structure predictions of nine genotypes were merged and displayed in different colors (d). Alignment of the loops in VP1 of nine prototype strains of enteroviruses (e). Codons of the loops in panel B, C and E was referred to EV-A71 (BrCr) genome: BC loop (94-107), DE loop (141-149), EF loop (158-177), GH loop (193-231), HI loop (237-245). Dot line: marker of 60%; ∗ Consistent codon.

### 3.4 VP1-based BEAST analysis

To investigate evolutionary history, maximum clade credibility (MCC) trees were constructed using complete VP1 sequences from representative strains of each serotype (Supplementary Fig. S4–S12). The estimated nucleotide substitution rate for the VP1 gene ranged from 2.76 × 10^−3^ (95% HPD, 2.57 × 10^−3^-2.96 × 10^−3^) to 4.29 × 10^−3^ (95% HPD, 3.95 × 10^−3^-4.65 × 10^−3^) substitutions per site per year (Fig. 4a). The time to the most recent common ancestor (tMRCA) for most viruses was estimated around the 1950s, consistent with their initial identification. However, the tMRCA for CVB5 was dated to 1859 (95% HPD: 1874.8–1947.4), significantly earlier than others (Fig. 4b), likely due to greater genetic divergence between its genotypes A and B (Supplementary Fig. S10). MCC trees revealed that most viruses diverged into two major branches early in their evolution and maintained low diversity until the late 1990s, as reflected in their Bayesian skyline plots (Fig. 4c). Over the subsequent 20–30 years, these viruses differentiated into multiple lineages with varying substitution rates (Supplementary Fig. S4–S12). Some lineages showed geographical specificity; for example, most C4 genogroup EV-A71 (C4-EV-A71), D-CVA2, E-CVA4, D3-CVA6, C-CVA10, B1-CVA16, D-CVB3, and B-CVB5 strains were isolated in China (Supplementary Fig. S4–S11), indicating local circulation and dominance. In contrast, CVA10, CVA16, and CVB3 strains from India belonged to genogroups E, F, B1b, and C, respectively, highlighting genetic distinctions between these two major Asian countries. For EV-D68, no distinct country- or region-specific clustering was observed (Supplementary Fig. S12), suggesting potential international spread, possibly originating from the USA. Therefore, enhanced molecular surveillance for EV-D68 outside the USA is warranted.

According to the Bayesian skyline plot analysis, we observed different trends in the relative genetic diversity (RGD) of the VP1 gene among these viruses (Fig. 4c). The RGD of most viruses (CVA2, CVA4, CVA10, CVB3 and CVB5) remained relatively stable before 2000, increased rapidly between 2000 and 2010, and then declined steadily (Fig. 4c). The RGD of the dominant viruses such as EV-A71 and CVA16 began increasing in the 1990s and continued to rise; a similar pattern was observed for CVA6, which became globally dominant after 2010. EV-D68 RGD showed two minor peaks around 2015, corresponding to submission data (Fig. 1a), followed by a sharp decline in 2020, reaching minimal genetic polymorphism (Fig. 4c). A subsequent V-shaped recovery indicated a rapid resurgence. These findings suggest that global anti-COVID-19 NPIs were effective in temporarily reducing respiratory virus transmission.

### 3.5 Characteristics of nucleotide and amino acid mutations in VP1

The outer capsid protein VP1 contains critical antigenic epitopes and is widely used for genotyping and phylogenetic studies. Thus, *VP1* variation is a key indicator of viral evolution and transmission. To assess cumulative mutation rates, we compared nucleotide mutations (NtMs) with non-synonymous amino acid substitutions (NSASs) in VP1. Notably, cumulative NSASs surpassed corresponding NtMs in the three dominant serotypes (EV-A71, CVA16, CVA6) around 2010, suggesting that high prevalence drives increased NSASs (Fig. 5a). We observed that this “transcendence” phenomenon occurred when cumulative NtMs reached approximately 60%; subsequently, cumulative NSASs could exceed 90% for EV-A71 and CVA16. Similarly, the NSAS rate in EV-D68 strains tended to exceed the NtM rate, consistent with its recurrent outbreaks in North America and elsewhere (Fig. 5a and. 1a). Based on the “60% transcendence” observation, increased molecular monitoring of VP1 NtMs in CVA4, CVA10, CVB3, and CVB5 is recommended. Further analysis of amino acid variability indicated that the e N- and C-terminal regions of VP1 were more variable than other regions (Fig. 5b). We focused on five well-characterized capsid loops in VP1: BC, DE, EF, GH, and HI (Fig. 5c and d). Most enterovirus neutralizing epitopes are located within these loops as conformational or linear epitopes; their amino acid variability is shown in Fig. 5b and e Among these loops, the nine serotypes shared the highest amino acid identity within the EF and GH loops (Fig. 5e). Shannon entropy analysis of VP1 amino acids for each serotype further reflected site-specific variability (Supplementary Fig. S13). Moreover, a strong correlation between VP1 NtMs and NSASs was observed for all serotypes, with Pearson correlation coefficients (*r*) ranging from 0.8772 (95% CI: 0.6751–0.9569) for CVA2 to 0.9766 (95% CI: 0.9447–0.9902) for EV-D68 (Supplementary Fig. S14).

Selection pressure analysis using MEME revealed episodic positive (diversifying) selection at several amino acid positions within VP1, including sites in the BC, DE, EF, GH, and HI loops across different serotypes (Table 1). This finding suggests ongoing selective pressure on VP1 neutralizing epitopes and underscores the need for continuous molecular surveillance of enterovirus evolution.

**Table 1:**
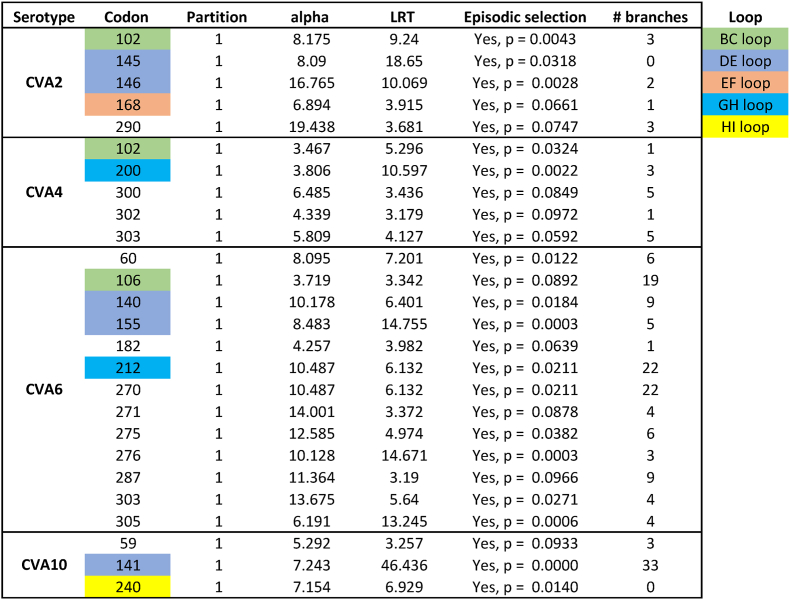

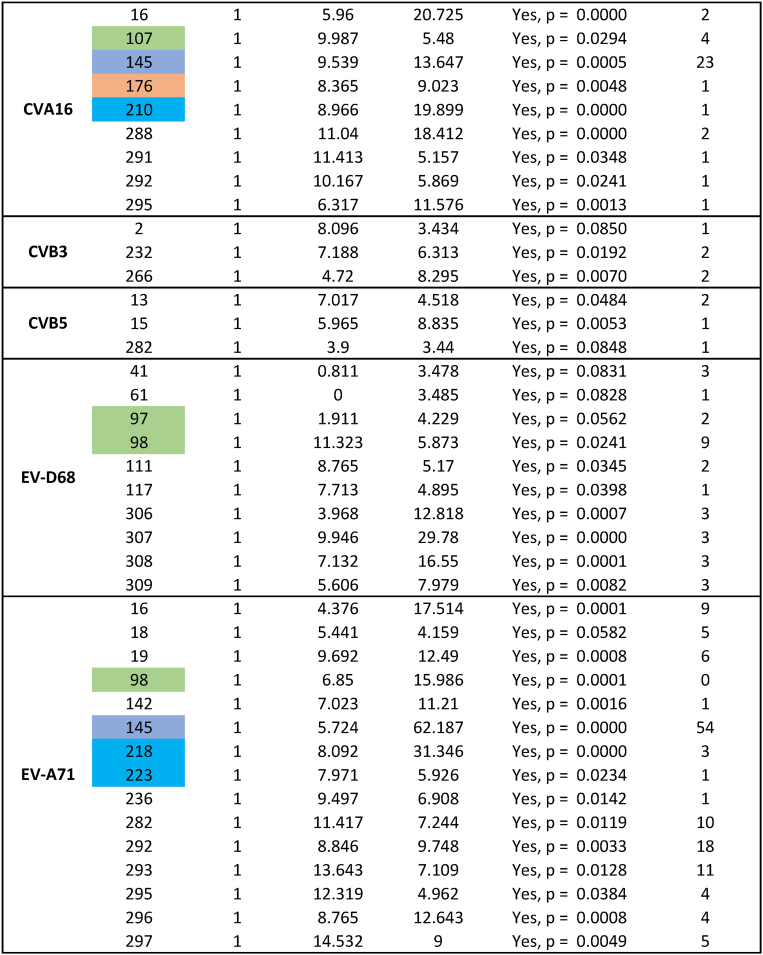
Selection pressure analysis of viral VP1 using MEME model in HyPhy software.

## 4 Discussion

Enteroviruses cause a broad spectrum of human diseases, from mild respiratory infections to severe conditions such as meningitis and encephalitis [4]. In recent years, concerns have grown regarding their mutation, recombination potential, and virulence [[13], [14], [15]]. Evolutionary flexibility has enabled enteroviruses to spread rapidly and establish themselves in diverse populations worldwide [16,17]. Substantial fluctuations in the pathogen spectrum have been observed across WHO regions, particularly with the rise of CVA6 and EV-D68 over the past decade (Fig. 2) [18,19]. Global epidemic trend of enteroviruses can be attributed to factors such as increased international travel, urbanization and climate change [20], which create favorable conditions for transmission, emergence of new strains, and re-emergence of existing ones. These processes generate viral variants with altered biological properties, including potentially increased virulence or antiviral resistance, posing challenges for vaccine development. In this study, we preliminarily describe a “60% Transcendence” phenomenon, which may serve as a useful marker for monitoring VP1 NSASs. Notably, NSASs appear concentrated within capsid loops, likely reflecting selective pressure on these neutralizing epitopes. High NSAS rates in these regions could facilitate immune evasion.

Based on submitted data, we observed periodic shifts in the regional and global pathogen spectrum, consistent with previous reports. From 2005 onwards, EV-A71, CVA16 and CVB5 were globally dominant, particularly in Europe (EV-A71 and CVB5), the Eastern Mediterranean (CVB5), and the Asia-Pacific region (EV-A71 and CVA16). Between 2005 and 2010, outbreaks became more frequent, and the pathogen spectrum fluctuated markedly as emerging/re-emerging strains like CVA6 began to dominate in several European countries. This CVA6-dominant trend persisted in Europe and spread globally over the following decade, accompanied by frequent recombination events [15,[21], [22], [23]]. Concurrently, regional EV-D68 outbreaks began in 2014, exhibiting a biennial peak and posing a threat to children's health, especially those with asthma, in the absence of a vaccine [5,24]. Developing highly effective vaccines is challenging due to pathogen spectrum fluctuations, mutation, and recombination [25]. However, nationwide vaccination in China has successfully controlled EV-A71 epidemics, albeit with subsequent shifts in the circulating pathogen spectrum [12,26]. Significant progress has been made in the past decade in developing multivalent vaccines against prevalent genotypes [27,28] and mRNA vaccine platforms [29]. Nevertheless, continuous mutation of antigenic epitopes and frequent recombination in non-structural protein genes present substantial challenges for clinical trials and regulatory approval. Additional hurdles include preclinical safety assessment, chemistry/manufacturing/controls (CMC), and the phased clinical trial process (Phases I–III). Until vaccines become widely available, public health measures—such as promoting hand hygiene and mask-wearing among preschool children during peak enterovirus seasons—remain crucial. Furthermore, wastewater surveillance could be implemented to monitor virus circulation, track pathogen spread, provide genome-based transmission insights, and guide future outbreak preparedness and response [[30], [31], [32], [33]].

Although this study provides a comprehensive analysis of the global distribution and evolution of key enterovirus serotypes, certain limitations should be acknowledged. 1) Submission data are inherently limited in volume and continuity compared to surveillance data, which may result in a higher proportion of residuals in time series decomposition; 2) Sequencing biases exist due to variable resource availability, local sequencing capacity, and the subset of symptomatic cases that reach healthcare facilities. A vast amount of global enterovirus surveillance data remains unpublished or unreleased in GenBank, limiting the completeness of VP1-based evolutionary analyses; 3) Results from Bayesian skyline plots can be influenced by sampling density, highlighting the importance of both the number and geographical representativeness of sequenced strains. To overcome these limitations, concerted efforts are needed to establish a real-time data-sharing network, exemplified by platforms like NextStrain and GISAID. Such an initiative must be founded on continuous molecular surveillance of enteroviruses from diverse sources, including clinical specimens, environmental samples, and wastewater.

In conclusion, understanding the global epidemic trends and evolutionary characteristics of enteroviruses is crucial for developing effective prevention and control strategies. Key efforts include advancing vaccine and antiviral drug development and implementing public health interventions to limit the spread of enteroviruses and reduce their burden on global health systems.

## Funding

This work was supported by the National Natural Science Foundation of China [Grant No. 32360003], the Research Start-up Fund of Jiangxi University of Chinese Medicine [Grant No. 2023BSZR001], Jiangxi University of Chinese Medicine National-Level College Student Innovation and Entrepreneurship Training Program [Grant No. 202510412001], and Jiangxi Provincial Health Commission Science and Technology Planning Project [Grant No. 202510742, 2026L1010].

## CRediT authorship contribution statement

**Han Mo:** Data curation, Formal analysis, Investigation, Methodology, Resources, Software, Validation, Visualization, Writing – review & editing. **Hui Li:** Data curation, Investigation, Project administration, Supervision, Writing – review & editing. **Jiadong Wu:** Data curation, Investigation, Methodology, Software, Visualization. **Liu Yi:** Formal analysis, Resources, Validation, Visualization. **Fenglan He:** Data curation, Formal analysis, Resources, Validation, Visualization, Writing – review & editing. **Qingmei Huang:** Formal analysis, Methodology, Resources, Validation, Visualization, Writing – review & editing. **Xian Zhang:** Data curation, Investigation, Methodology, Resources, Validation, Writing – review & editing. **Qian Yang:** Formal analysis, Investigation, Project administration, Supervision, Visualization, Writing – review & editing. **Tianmu Chen:** Investigation, Resources, Supervision, Validation, Writing – review & editing. **Xianfeng Zhou:** Conceptualization, Data curation, Formal analysis, Funding acquisition, Investigation, Methodology, Project administration, Resources, Software, Supervision, Validation, Visualization, Writing – original draft, Writing – review & editing.

## Declaration of competing interest

The authors declare that they have no known competing financial interests or personal relationships that could have appeared to influence the work reported in this paper.

## Data Availability

Data will be made available on request.
