# Supplementary material for: Global distribution and evolution of nine major non-polio enteroviruses revealed by genomic data mining
Source: Biochem Biophys Rep. 2026 Feb 9;45:102485. doi: 10.1016/j.bbrep.2026.102485 (PMC12907910; doi:10.1016/j.bbrep.2026.102485)

**Supplementary Figure S1**

**A five-year interval time course of accumulated composition of nine serotypes CVA2, CVA4, CVA6, CVA10, CVA16, CVB3, CVB5, EV-A71 and EV-D68 around the world by 2023.** The first time point was set in 2000 as very limited submission by 2000. Accumulated data was shown for each time point.


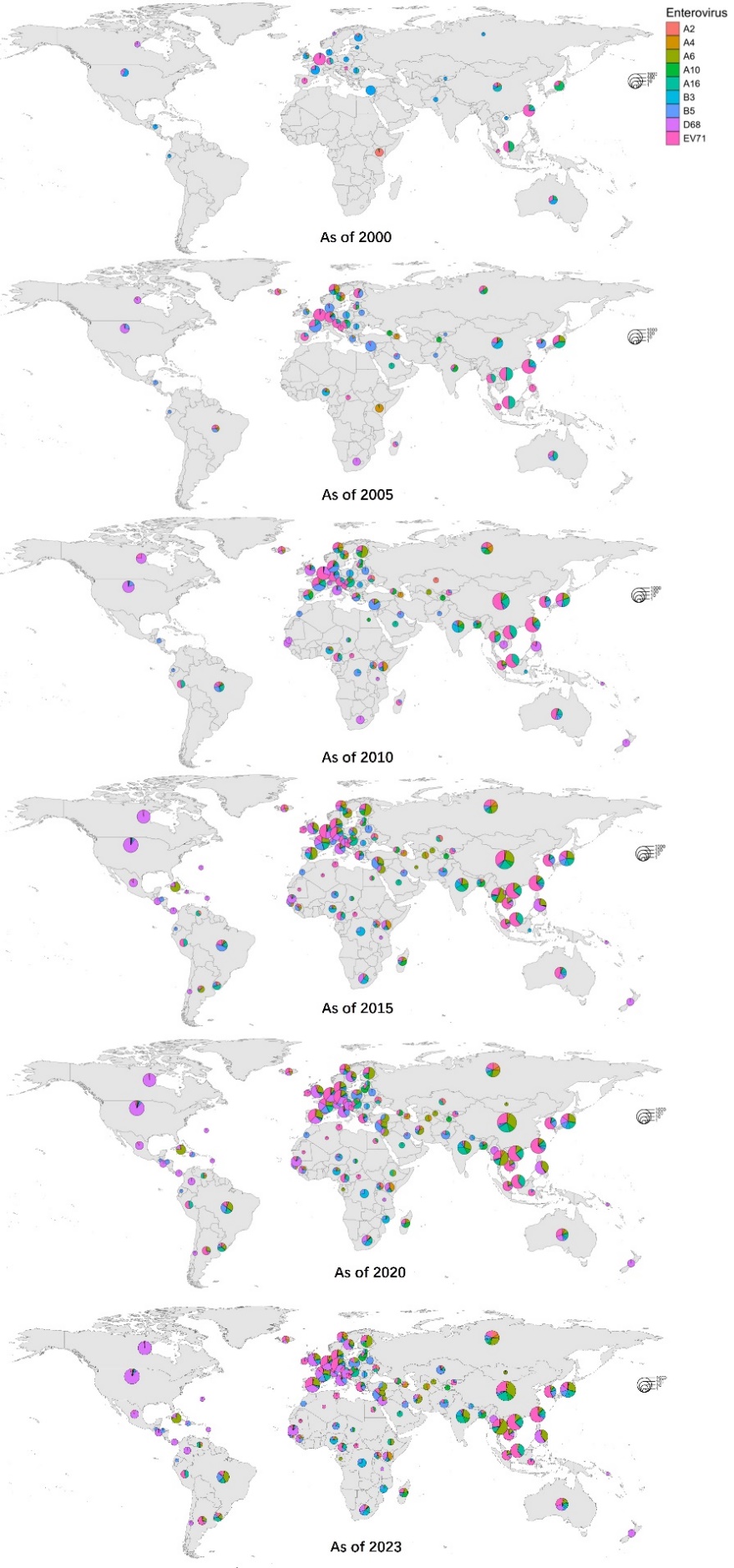


**Supplementary Figure S2**

Seasonal fitting of viral epidemics of three viruses CVA10, CVA16 and EV-A71 using submission data from 2010 to 2019.


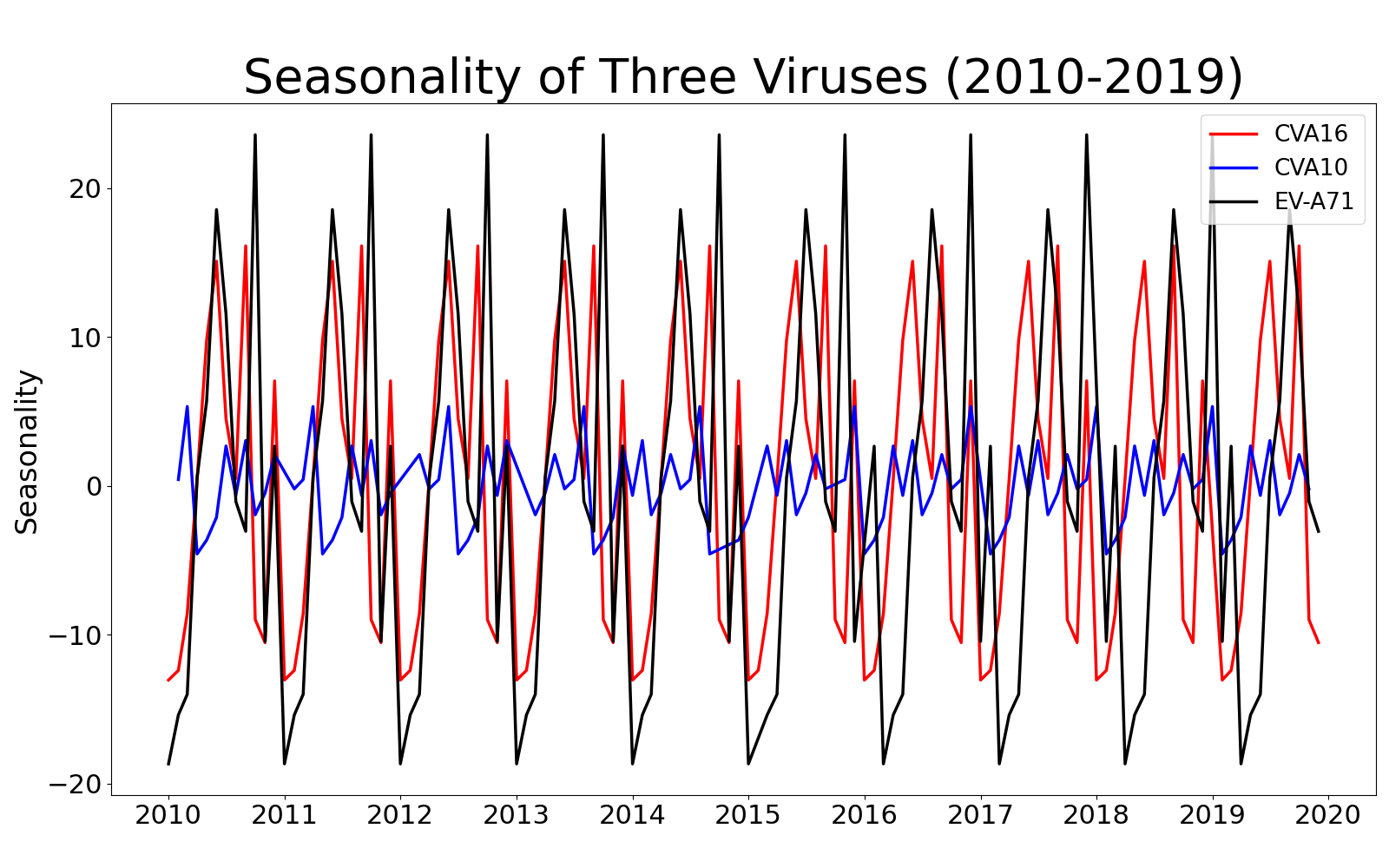


**Supplementary Figure S3**

Time series decomposition in trend, seasonality, and residuals of each serotype. A) Global submission data from 2010-2023; B) data without strains from China. Curves of count, trend, seasonal and residua were displayed for each serotype.

A


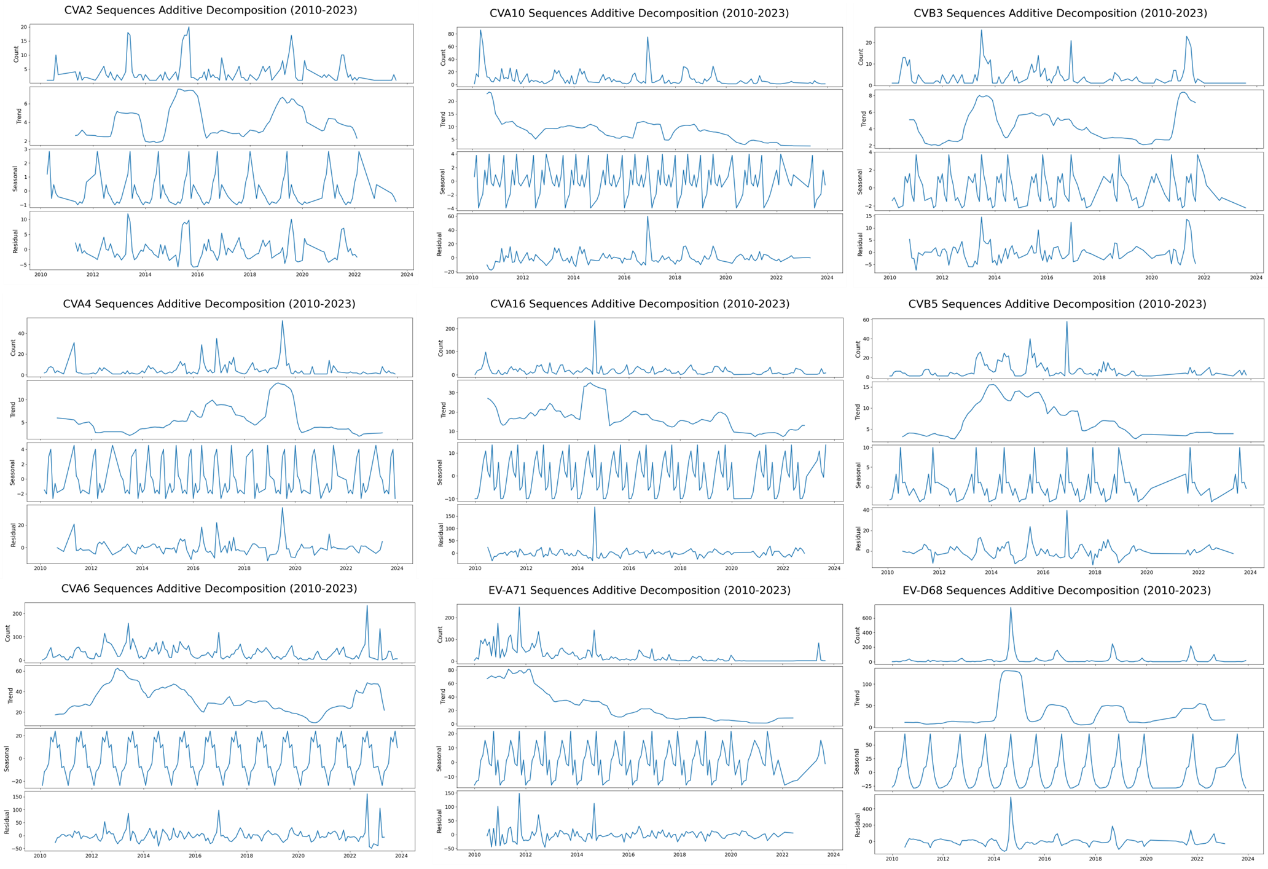


B


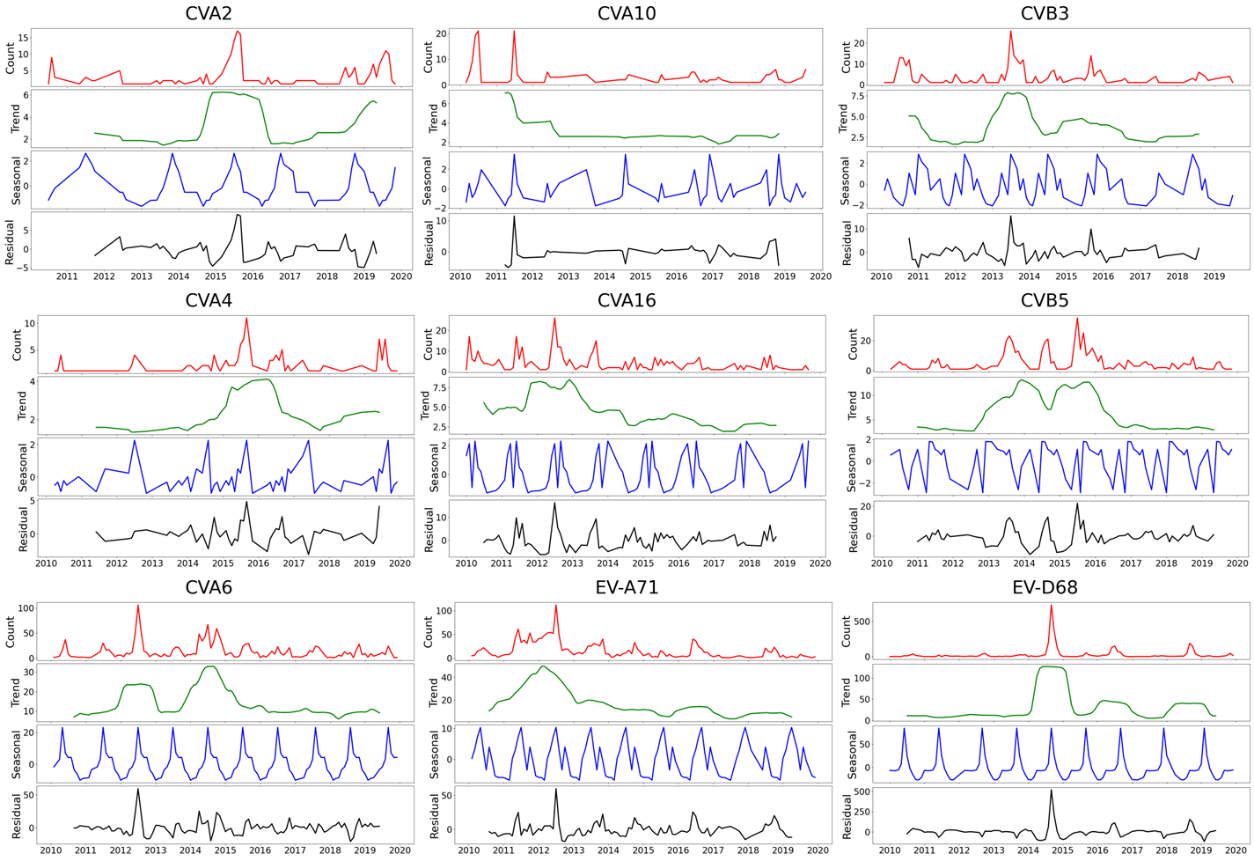


**Supplementary Figure S4**

Maximum clade credibility (MCC) tree of the representative CVA2 strains (n=196). MCC tree derived from the Bayesian analysis of the VP1 protein of CVA2 with the best fit model (strict molecular clock model), showing the time to the most recent common ancestor (tMRCA) in each principal node. Country with less than 5 representative strains were labeled as other country.


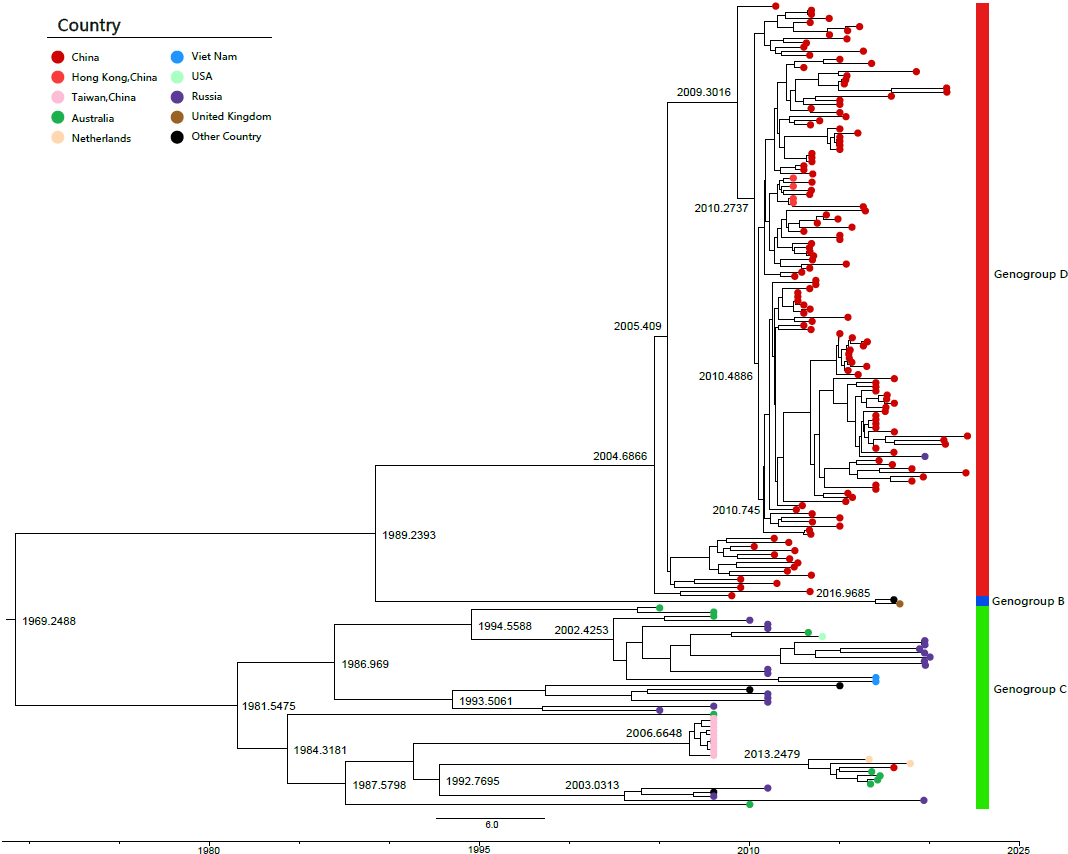


**Supplementary Figure S5**

Maximum clade credibility (MCC) tree of the representative CVA4 strains (n=191). MCC tree derived from the Bayesian analysis of the VP1 protein of CVA4 with the best fit model (strict molecular clock model), showing the time to the most recent common ancestor (tMRCA) in each principal node.


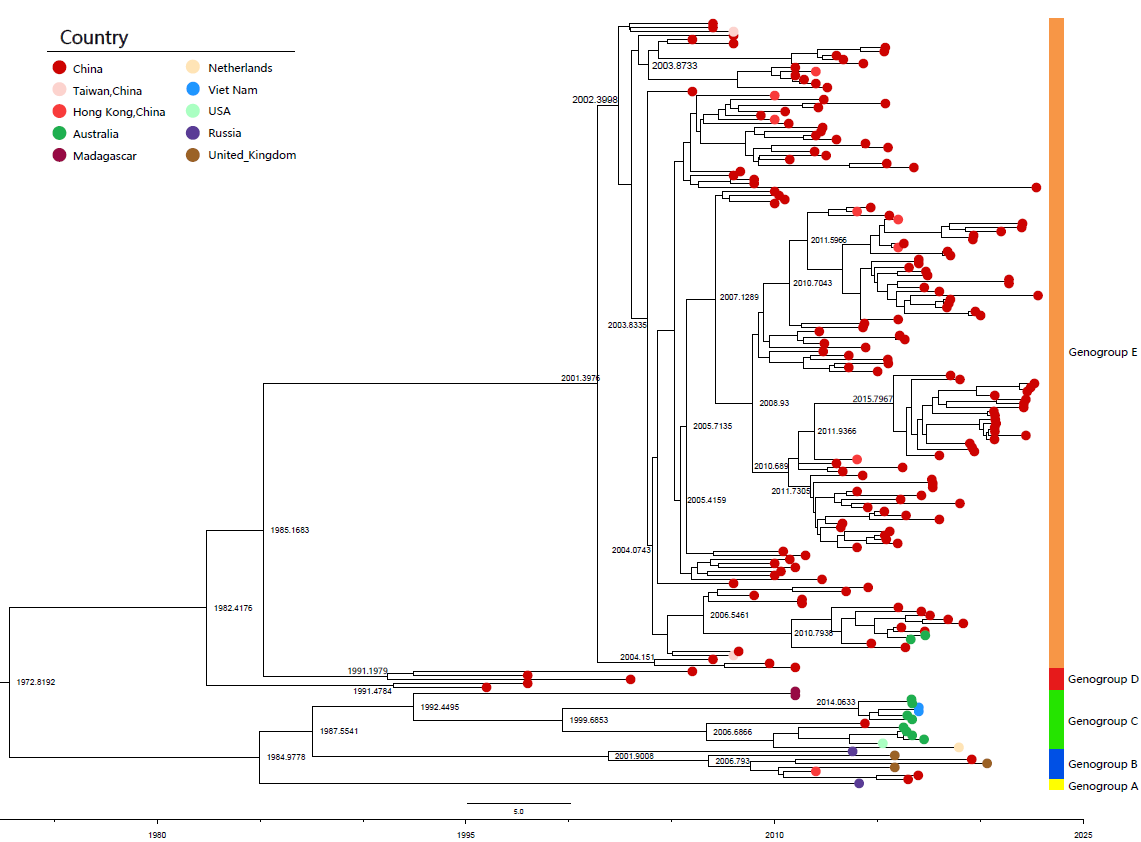


**Supplementary Figure S6**

Maximum clade credibility (MCC) tree of the representative CVA6 strains (n=309). MCC tree derived from the Bayesian analysis of the VP1 protein of CVA6 with the best fit model (strict molecular clock model), showing the time to the most recent common ancestor (tMRCA) in each principal node. Country with less than 5 representative strains were labeled as other country.


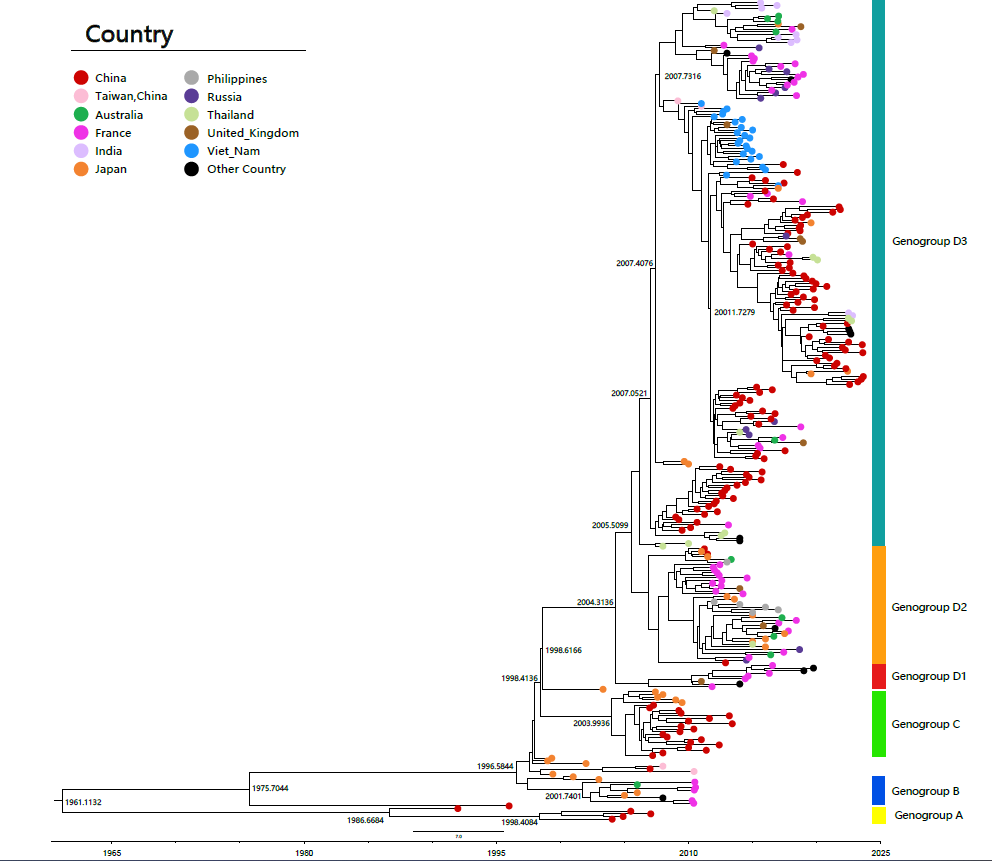


**Supplementary Figure S7**

Maximum clade credibility (MCC) tree of the representative CVA10 strains (n=216). MCC tree derived from the Bayesian analysis of the VP1 protein of CVA10 with the best fit model (strict molecular clock model), showing the time to the most recent common ancestor (tMRCA) in each principal node. Country with less than 5 representative strains were labeled as other country.


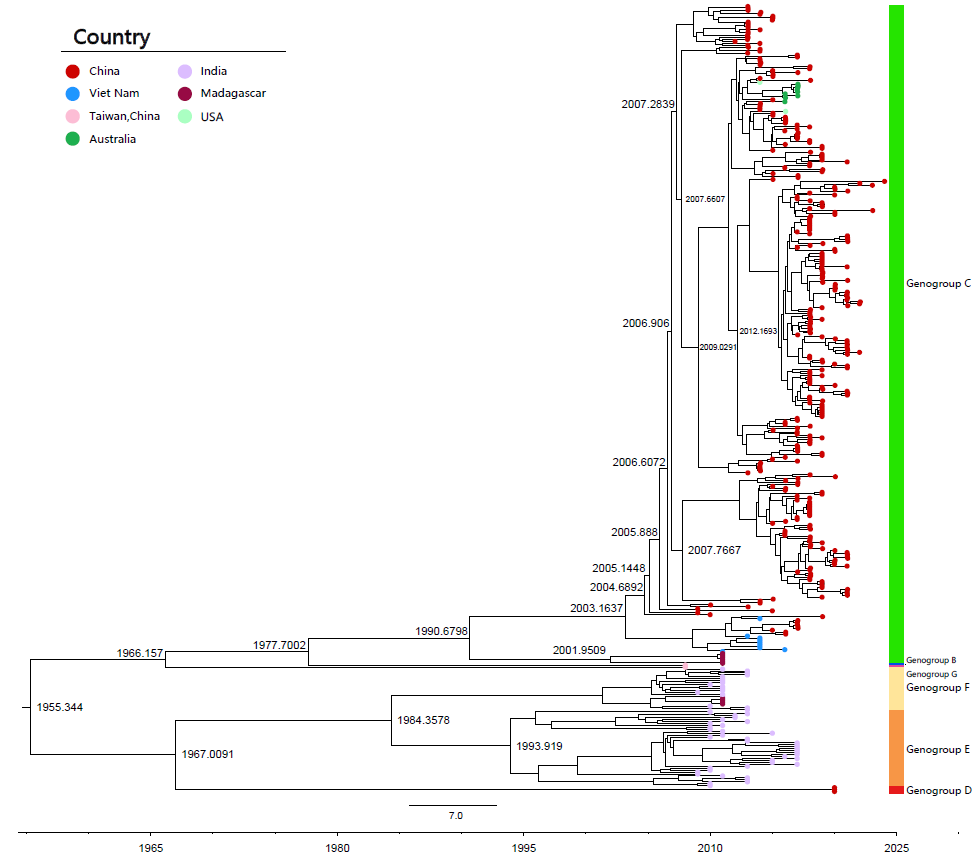


**Supplementary Figure S8**

Maximum clade credibility (MCC) tree of the representative CVA16 strains (n=223). MCC tree derived from the Bayesian analysis of the VP1 protein of CVA16 with the best fit model (strict molecular clock model), showing the time to the most recent common ancestor (tMRCA) in each principal node. Country with less than 5 representative strains were labeled as other country.


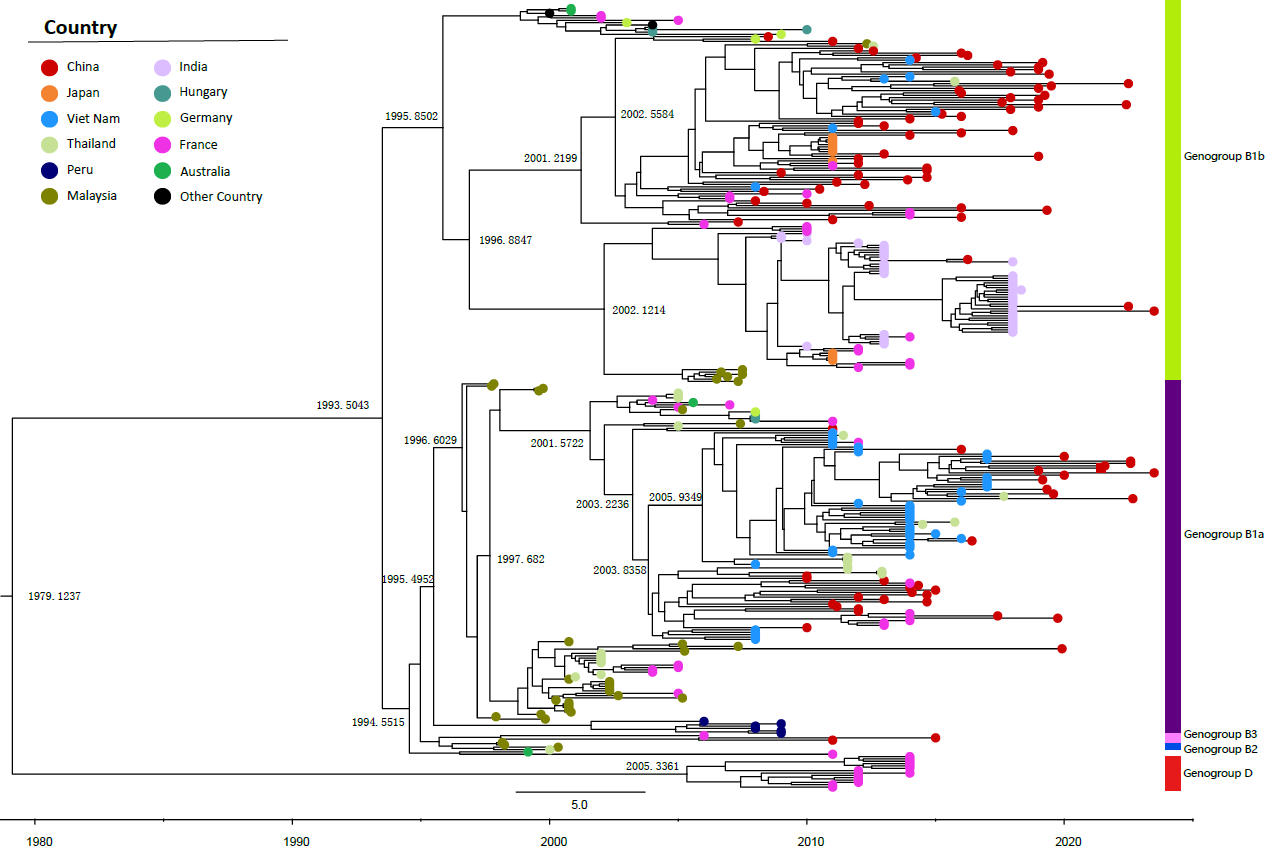


**Supplementary Figure S9**

Maximum clade credibility (MCC) tree of the representative CVB3 strains (n=188). MCC tree derived from the Bayesian analysis of the VP1 protein of CVB3 with the best fit model (strict molecular clock model), showing the time to the most recent common ancestor (tMRCA) in each principal node. Country with less than 5 representative strains were labeled as other country.


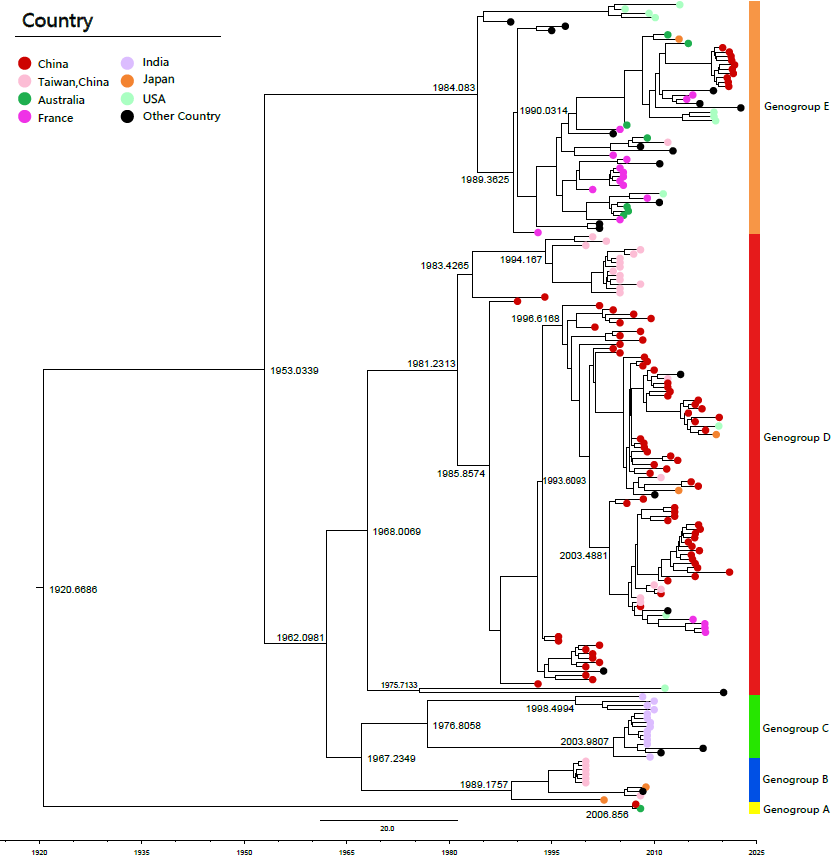


**Supplementary Figure S10**

Maximum clade credibility (MCC) tree of the representative CVB5 strains (n=263). MCC tree derived from the Bayesian analysis of the VP1 protein of CVB5 with the best fit model (strict molecular clock model), showing the time to the most recent common ancestor (tMRCA) in each principal node. Country with less than 5 representative strains were labeled as other country.


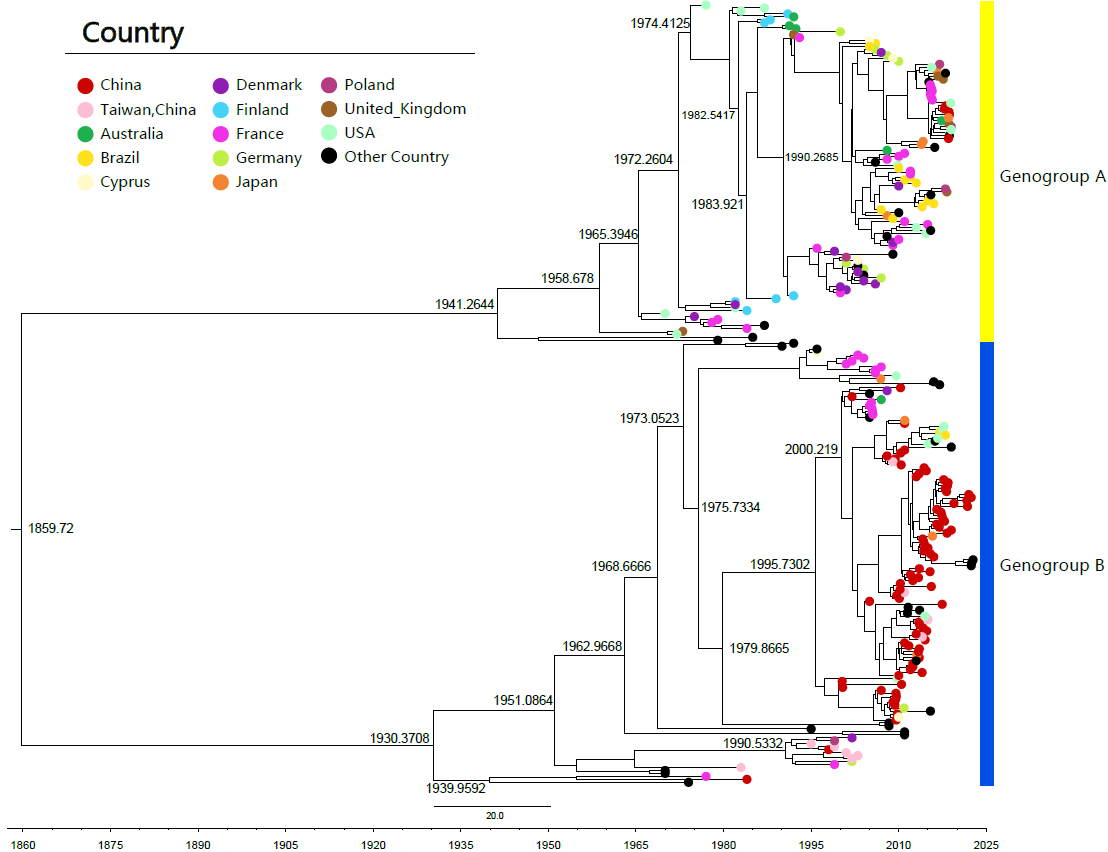


**Supplementary Figure S11**

Maximum clade credibility (MCC) tree of the representative EV-A71 strains (n=305). MCC tree derived from the Bayesian analysis of the VP1 protein of EV-A71 with the best fit model (strict molecular clock model), showing the time to the most recent common ancestor (tMRCA) in each principal node. Country with less than 5 representative strains were labeled as other country.


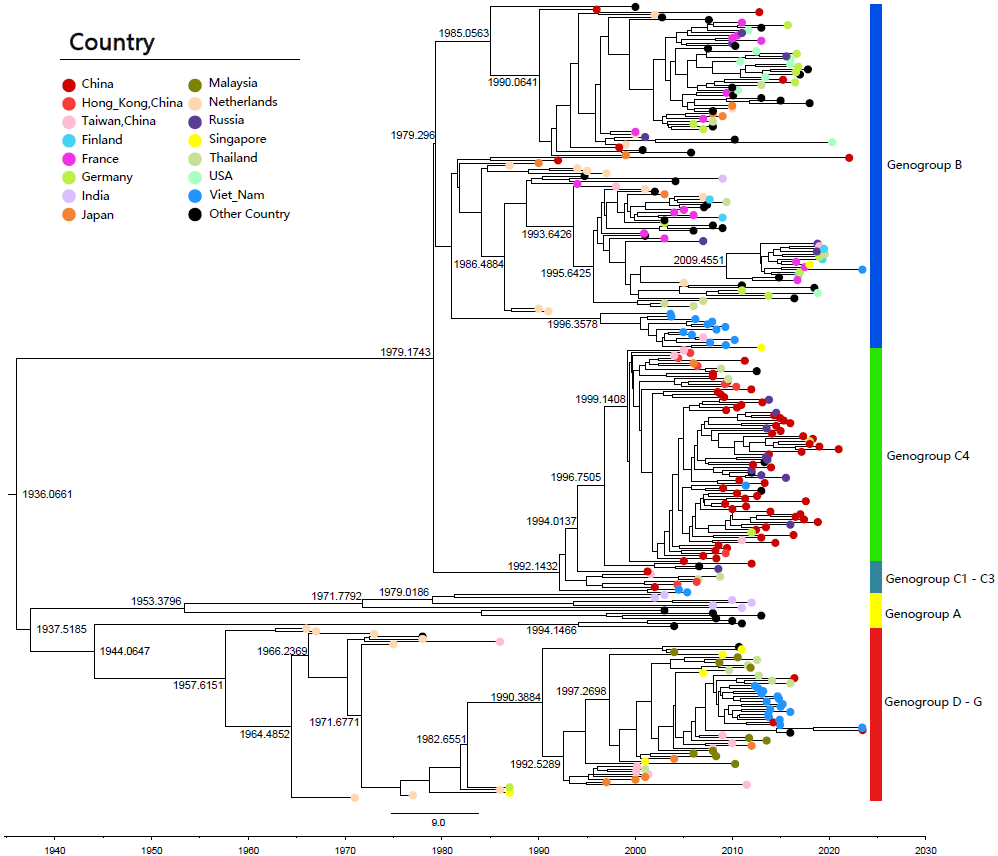


**Supplementary Figure S12**

Maximum clade credibility (MCC) tree of the representative EV-D68 strains (n=294). MCC tree derived from the Bayesian analysis of the VP1 protein of EV-D68 with the best fit model (strict molecular clock model), showing the time to the most recent common ancestor (tMRCA) in each principal node. Country with less than 5 representative strains were labeled as other country.


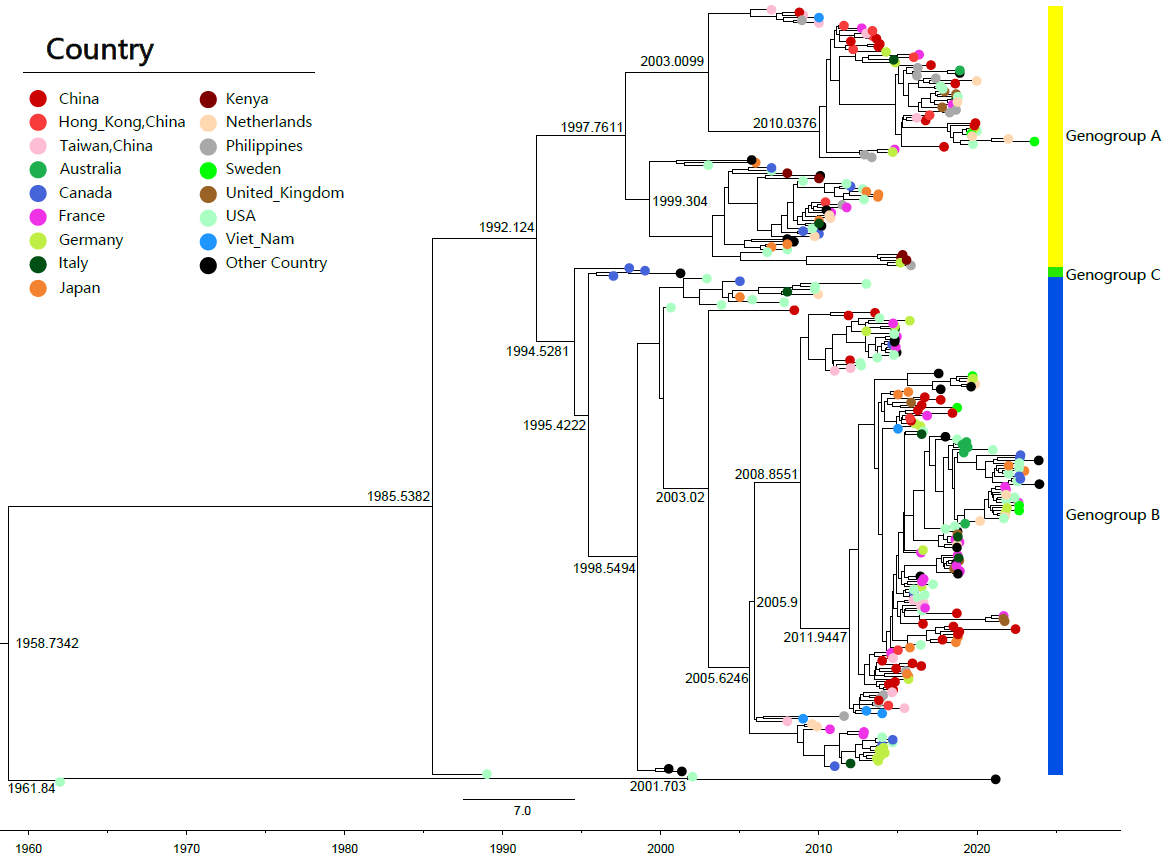


**Supplementary Figure S13**

Shannon entropy of VP1 amnio acid sites of each serotype


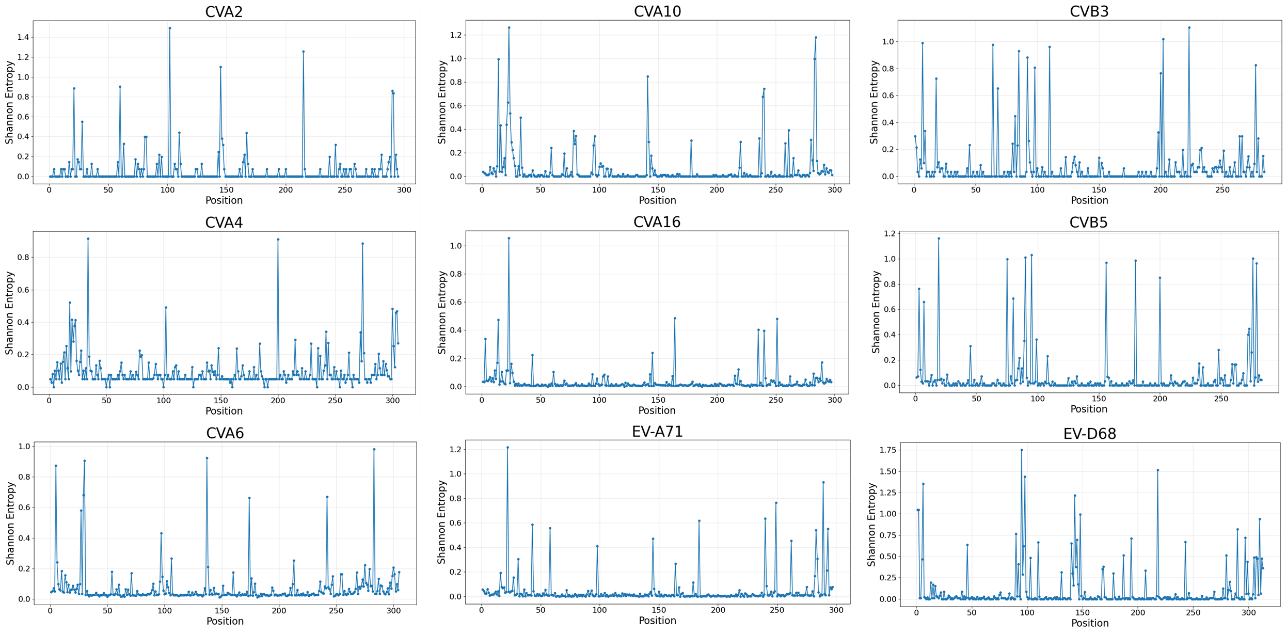


**Supplementary Figure S14**

**Correlation of cumulative nucleotide (nt) mutations and non-synonymous mutation of amino acids (aa) of each serotype using Pearson correlation analysis.**


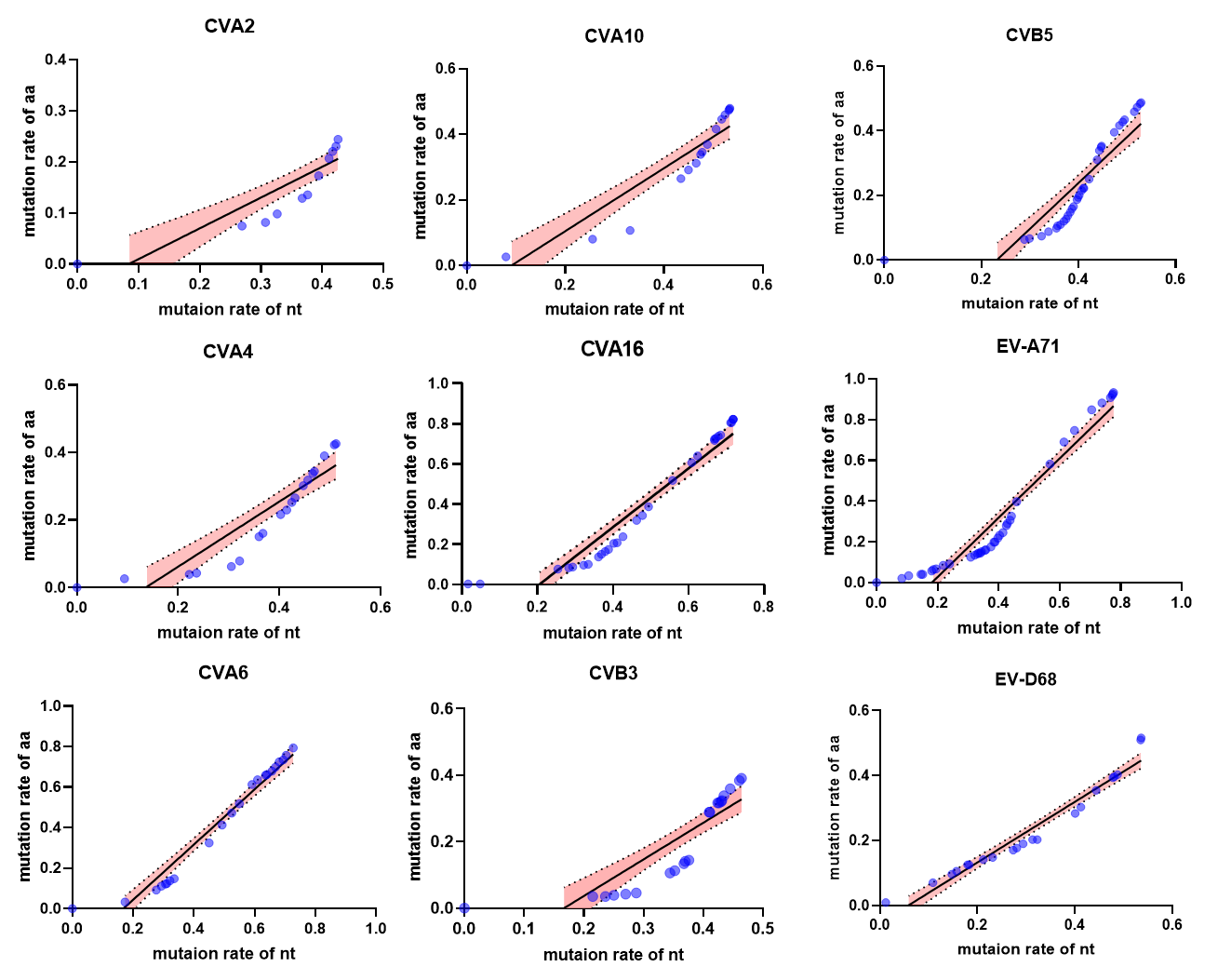

Supplement: Multimedia component 1 [file mmc1.docx]
